# The PRO-ACTIVE trial protocol: a randomized study comparing the effectiveness of PROphylACTic swallow InterVEntion for patients receiving radiotherapy for head and neck cancer

**DOI:** 10.1186/s12885-021-08826-0

**Published:** 2021-10-13

**Authors:** R. Martino, M. I. Fitch, C. D. Fuller, A. Hope, G. Krisciunas, S. E. Langmore, C. Lazarus, C. L. Macdonald, T. McCulloch, G. Mills, D. A. Palma, K. Pytynia, J. Ringash, K. Sultanem, J. Theurer, K. E. Thorpe, K. Hutcheson

**Affiliations:** 1 Department of Speech Language Pathology, University of Toronto, 160-500 University Ave, Toronto, Ontario M5G 1V7 Canada; 2 Rehabilitation Science Institute, University of Toronto, 160-500 University Ave, Toronto, Ontario M5G 1V7 Canada; 3 Krembil Research Institute, University Health Network, Toronto, Ontario Canada; 4 Department of Otolaryngology, University of Toronto, 160-500 University Ave, Toronto, Ontario M5G 1V7 Canada; 5 Bloomberg Faculty of Nursing, University of Toronto, Toronto, Ontario Canada; 6 Division of Radiation Oncology, University of Texas M.D. Anderson Cancer Center, 7007 Bertner Ave, Houston, TX TX 77030 USA; 7 Department of Radiation Oncology, University of Toronto, Toronto, Ontario Canada; 8 Department of Radiation Oncology, Princess Margaret Hospital/University Health Network, Toronto, Ontario Canada; 9 Department of Otolaryngology-Head & Neck Surgery, Boston University School of Medicine, Boston, USA; 10 Department of Otolaryngology - Head and Neck Surgery, Mount Sinai Beth Israel, New York, NY USA; 11 Qualitative Health Research Consultants, Madison, WI USA; 12 Department of Surgery, Division of Otolaryngology - Head & Neck Surgery, University of Wisconsin-Madison, Madison, WI U.S.A.; 13 Department of Radiation Oncology, McGill University, Montreal, QC Canada; 14 Department of Radiation Oncology, Western University, London, ON Canada; 15 Department of Head & Neck Surgery, University of Texas MD Anderson Cancer Center, 7007 Bertner Ave, Houston, TX TX 77030 USA; 16 School of Communication Sciences and Disorders, Western University, London, ON Canada; 17 Dalla Lana School of Public Health, University of Toronto, Toronto, ON Canada; 18 Applied Health Research Centre of the Li Ka Shing Knowledge Institute, Toronto, Canada

**Keywords:** Head and neck cancer survivors, Dysphagia, Intervention, Prophylactic, Reactive, Feeding tube duration

## Abstract

**Background:**

Swallowing therapy is commonly provided as a treatment to lessen the risk or severity of dysphagia secondary to radiotherapy (RT) for head and neck cancer (HNC); however, best practice is not yet established. This trial will compare the effectiveness of prophylactic (high and low intensity) versus reactive interventions for swallowing in patients with HNC undergoing RT.

**Methods:**

This multi-site, international randomized clinical trial (RCT) will include 952 adult patients receiving radiotherapy for HNC and who are at high risk for post-RT dysphagia. Participants will be randomized to receive one of three interventions for swallowing during RT: RE-ACTIVE, started promptly if/when dysphagia is identified; PRO-ACTIVE EAT, low intensity prophylactic intervention started before RT commences; or, PRO-ACTIVE EAT+EXERCISE, high intensity prophylactic intervention also started before RT commences. We hypothesize that the PRO-ACTIVE therapies are more effective than late RE-ACTIVE therapy; and, that the more intensive PRO-ACTIVE (EAT + EXERCISE) is superior to the low intensive PRO-ACTIVE (EAT). The primary endpoint of effectiveness is duration of feeding tube dependency one year post radiation therapy, selected as a pragmatic outcome valued equally by diverse stakeholders (e.g., patients, caregivers and clinicians). Secondary outcomes will include objective measures of swallow physiology and function, pneumonia and weight loss, along with various patient-reported swallowing-related outcomes, such as quality of life, symptom burden, and self-efficacy.

**Discussion:**

Dysphagia is a common and potentially life-threatening chronic toxicity of radiotherapy, and a priority issue for HNC survivors. Yet, the optimal timing and intensity of swallowing therapy provided by a speech-language pathologist is not known. With no clearly preferred strategy, current practice is fraught with substantial variation. The pragmatic PRO-ACTIVE trial aims to specifically address the decisional dilemma of when swallowing therapy should begin (i.e., before or after a swallowing problem develops). The critical impact of this dilemma is heightened by the growing number of young HNC patients in healthcare systems that need to allocate resources most effectively. The results of the PRO-ACTIVE trial will address the global uncertainty regarding best practice for dysphagia management in HNC patients receiving radiotherapy.

**Trial registration:**

The protocol is registered with the US Patient Centered Outcomes Research Institute, and the PRO-ACTIVE trial was prospectively registered at ClinicalTrials.gov, under the identifier NCT03455608; First posted: Mar 6, 2018; Last verified: Jun 17, 2021.

**Protocol Version:** 1.3 (January 27, 2020).

## Background

Dysphagia is a priority issue in Head and Neck cancer (HNC) survivorship. Difficulty swallowing (dysphagia) is a common and potentially life-threatening chronic toxicity of radiotherapy (RT) for HNC. While an independent predictor of quality of life (QOL) in HNC survivorship, dysphagia can also have a devastating impact on the health of patients. The lifetime risk of aspiration pneumonia is 20–24% after chemoradiation for HNC [1, 2], representing a 2.7-fold elevated risk of pneumonia in HNC survivors (age ≥ 65 years) over matched non-cancer controls in the United States population. Aspiration pneumonia also confers a 42% excess risk of mortality among HNC survivors who develop pneumonia relative to those who do not [1]. Moreover, dysphagia predisposes to malnutrition and nutritional deficiencies. Despite treatment advances, up to 70% require tube feeding, typically for a minimum of several months during and immediately following RT [3]; feeding tube dependency persists in approximately 45% of patients 3 months after finishing cancer treatment [4–9].

Despite improvements in conformal planning techniques to lessen normal tissue injury from RT [10], almost all patients experience some degree of early dysphagia. RT dose thresholds for dysphagia suggest that risk persists even with emerging investigational efforts in swallowing optimized RT planning [11]. While acute dysphagia improves for the majority of patients in the early months after RT ends, many survivors suffer chronic or progressive radiation-associated dysphagia years after treatment. In fact, among those with late (beyond 5 years) toxicities, 66% have severe dysphagia requiring lifelong feeding tube dependence [12]. Recent work highlights the severity of this endpoint to cancer survivors, who ranked feeding tube dependency as one of 6 outcomes of their cancer treatment that were worse than death [13].

There is a rapidly growing pool of HNC survivors at risk for chronic dysphagia**,** with 68,785 incident cases annually in the USA and Canada [14, 15]. A large proportion of HNCs are now human papillomavirus (HPV)-driven oropharyngeal cancers, the incidence of which is expected to sharply rise through 2030 [16]. Almost all of this fast growing, large subgroup of HNC survivors have been treated with curative RT at doses ≥60 Gray, sufficient to induce chronic dysphagia. HPV-associated HNC is diagnosed younger (median age: 54 years) [17] than tobacco-related HNC with excellent five-year survival probability of 79% [18]. As such, survivors have the potential to live many active years (even decades) with toxicities of RT including dysphagia.

Oncologists commonly refer patients for swallowing therapy with a speech language pathologist to lessen risk or severity of radiation-associated dysphagia, but best practice is not established using prospective evidence. Some patients are monitored and referred for dysphagia interventions *only in response to a swallow problem* (RE-ACTIVE therapy), a so-called “wait and see” approach. Thus, RE-ACTIVE therapy aims to reverse an already impaired swallow back to its functional pre-morbid state, typically with exercise and mealtime adjustment [19]. In contrast, other patients are referred pre-emptively for prophylactic “PRO-ACTIVE” swallowing therapy that aims to prevent or lessen the severity or duration of dysphagia. PRO-ACTIVE interventions are prescribed early – *before the swallow problem begins* – in an effort to inhibit disuse atrophy and fibrosis [19]. PRO-ACTIVE therapy can vary in intensity. Some receive PRO-ACTIVE therapy focused on keeping them eating to keep muscles active (our PRO-ACTIVE EAT group) [20], while others are also prescribed swallowing exercise between meals (our PRO-ACTIVE EAT+EXCERCISE group) [21]. The two broad categories, PRO-ACTIVE versus RE-ACTIVE, as well as the variation in intensity of PRO-ACTIVE therapy (‘EAT versus ‘EAT+EXERCISE’), represent the most common therapy models in North America [19] and the UK [22].

The benefits of swallowing therapy in ideal settings, regardless of its timing and intensity, are suggested by many single institution observational and controlled efficacy trials. Two recent systematic reviews (one from the PRO-ACTIVE investigators) showed benefits to various outcomes, including feeding tube dependence, QOL, swallow efficiency, function, and swallow physiology from both PRO-ACTIVE (early) and RE-ACTIVE (delayed) therapy [21, 23]. Specifically, pooled data from efficacy trials suggested lower feeding tube dependence among those who received PRO-ACTIVE therapy with exercise relative to either RE-ACTIVE or lower intensity PRO-ACTIVE therapy (odds ratio [OR]: 0.53, 90% CI: 0.29, 0.97, 21). Likewise, our meta-analysis identified equally convincing benefit to swallow function from RE-ACTIVE therapy (OR: 3.70, 95% CI, 1.53, 8.94) compared to either no therapy or less intensive therapy [21]. Unfortunately, no review to date has identified the timing (PRO-ACTIVE versus RE-ACTIVE) or intensity (less versus more) most effective when applied in a real-world setting. Despite several meritorious efforts using small efficacy trials and even more robust meta-analysis, the decisional dilemma regarding what is the best timing and intensity of behavioural swallowing interventions during RT remains, precluding evidence-based prescription and effective health system resource allocation.

The chief dilemma for therapy providers centers on *when* swallowing therapy should begin. The investigators’ survey [19] of North American speech language pathologists reported an even distribution among these therapies, with 48% of respondents practicing PRO-ACTIVE therapy versus 52% who used RE-ACTIVE therapy. Clearly clinical equipoise exists and contributes to variation in our practice. The dilemma is further heightened considering the target population comprises rapidly growing numbers of younger HNC survivors who face considerable competing toxicities during RT, of which dysphagia registers the among the most impactful [24]. This growing prevalence of HNC survivors with dysphagia makes it even more imperative to identify the most effective therapies, so that healthcare systems can allocate limited resources to optimize functional recovery after curative oncologic treatment.

## Aims and hypotheses

To address this evidence gap, the primary aim (Aim 1) of the PRO-ACTIVE trial is to compare the effectiveness of PRO-ACTIVE versus RE-ACTIVE swallowing interventions among patients with HNC planned to undergo RT. We hypothesize that at 12-months post radiotherapy the combined PRO-ACTIVE therapies are more effective than RE-ACTIVE therapy (Hyp 1.1); and, if so, that more intensive PRO-ACTIVE (EAT + EXERCISE) is superior to less intensive PRO-ACTIVE (EAT) (Hyp 1.2). Effectiveness will be measured based on reduced duration of feeding tube dependency, a pragmatic outcome valued by diverse stakeholders including patients, caregivers and clinicians [13, 21, 25, 26].

The second aim (Aim 2) of the PRO-ACTIVE trial is to compare the relative benefit or harm of PRO-ACTIVE to RE-ACTIVE swallowing intervention on all secondary outcomes at both 3- and 12- months post radiotherapy, namely: swallowing impairment, maximum interincisal opening (trismus), swallowing-related QOL, symptom burden, self-reported pneumonia, malnutrition and mood disorder, cancer coping strategies, diet level, feeding tube rates, weight loss, hospitalization and/or emergency department (ED) presentation. In addition, the second aim will compare the benefit on reduced duration of feeding tube presence at 3-month post radiotherapy. Our hypothesis for this second aim is that the combined PRO-ACTIVE interventions will result in better secondary outcomes at 3- and 12- months following the completion of RT (Hyp 2.1). As in our primary aim, for each secondary outcome for which PRO-ACTIVE is superior, dose response of the PRO-ACTIVE arms will be examined hypothesizing the more intensive therapy as superior (Hyp 2.2).

The third aim (Aim 3) of the PRO-ACTIVE trial is to explore the relative benefit or harm of the PRO-ACTIVE and RE-ACTIVE swallowing interventions in relevant subgroups according to the following: age, tumor site, prior surgery, radiation dose, chemotherapy use, and prophylactic feeding tube use. Our hypothesis for this third aim is that all interventions will be more effective in the following higher risk subgroups: older age; non-HPV related cancer sites; prior surgery; higher radiation dose; chemotherapy (yes); and, prophylactic feeding tube insertion (yes).

## Methods/design

### Overview of trial design

This is a multi-center, single-blinded 3-arm behavioural intervention pragmatic RCT that will compare the effectiveness of PRO-ACTIVE versus RE-ACTIVE swallowing interventions in patients with HNC receiving RT across eleven sites in Canada and the United States. The PRO-ACTIVE trial design was developed using PRECIS-2 framework for pragmatic trial methodology and adheres to the funding agency, Patient Centered Outcomes Research Institute (PCORI), methodology standards [27, 28]. The research question was determined based on identification of practice variation with clinicians equally committed to each intervention [19, 22], and gap analysis from two recent systematic reviews from the investigative team [21] and others [23] establishing clinical equipoise between RE-ACTIVE and PRO-ACTIVE swallowing intervention. Further refinements to the trial protocol, including the selection of secondary outcomes, were made with input from patient and clinical stakeholders following PCORI guidelines for stakeholder engagement. The trial protocol is in accordance with the principles of the Declaration of Helsinki and has been approved by Research Ethics Boards across all sites. The trial was prospectively registered on Clinicaltrials.gov (ClinicalTrials.gov Identifier: NCT03455608). Any changes that need to be made in the trial protocol will be communicated to all investigators, the ethics committees, and the trial registry. Written informed consent will be obtained from each participant.

A total of 952 participants with HNC planned for RT who do not currently have dysphagia will be randomized to receive one of 3 swallowing interventions by speech language pathologists:.

- Arm 1. delayed intervention started promptly if/when dysphagia is identified (RE-ACTIVE)
- Arm 2. early low intensity intervention started before RT commences (PRO-ACTIVE EAT)
- Arm 3. early high intensity intervention started before RT commences (PRO-ACTIVE EAT+ EXERCISE)

**Fig. 1:**
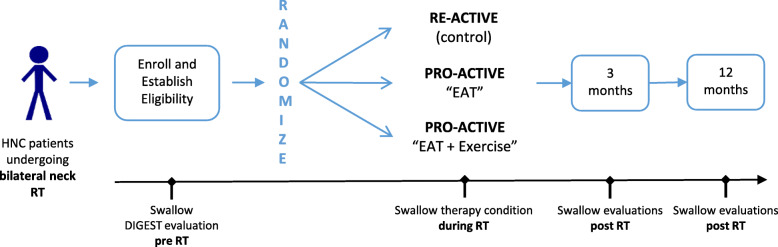
PRO-ACTIVE Study Schemata

The randomization of participants will be stratified by participating site using a centralized, automated and concealed process conducted by the Data Coordinating Center (DCC). Participants and therapy providers will not be blinded; however, clinical raters and data analysts will be blinded to the study arm to minimize bias. Eligible patients will be randomized in a 1:2:2 allocation to RE-ACTIVE (Arm 1, *n* = 190) and each of the PRO-ACTIVE arms (Arms 2 and 3, *n* = 381 per group). All patients will be followed to 12 months post-RT. The main intention to treat analysis will compare duration of feeding tube dependence post-RT. The study schemata is shown in Fig. 1.

### Study settings

Cancer treating centers will be included across eleven North American cities to account for possible regional variation (Toronto, Houston, Boston, Madison, New York, London, Montreal, Baltimore, Detroit Miami, Orlando). The Princess Margaret Cancer Centre, part of the University Health Network (PM/UHN) in Toronto, serves as the prime site. In the USA, where patients are as likely to receive treatment at an academic or community facility, we will aim to enroll 50% of participants from both academic and community-based facilities. In Canada, where cancer therapy is regionalized at academic teaching facilities, we will enroll all participants from representative academic centers but across two provinces thereby capturing regional variation.

### Study participants

Eligibility criteria were defined to allow participation of any patient for whom the following decisional dilemma exists: “is there a benefit to proactive swallowing therapy during RT?” In keeping with pragmatic trial standards, the eligibility criteria were operationalized to include any patient who presents to begin RT for HNC with a functional swallow but at high risk for developing post-RT dysphagia: defined as planned RT dose ≥60 Gy with treatment volumes including bilateral neck, based on published prediction models for tube-dependent dysphagia [29]. Our local registries confirm that these criteria should include more than 75% of patients who enter curative (primary or adjuvant) intent RT. As a pragmatic RCT, we will limit restrictiveness of eligibility, thereby seeking to include patients who are commonly excluded from efficacy trials. Specifically, common exclusions we have avoided for this pragmatic study are: prior surgery or RT; non-squamous cell histology; prior swallowing therapies unrelated to current HNC; and selected HNC sites and stages. We will enroll patients with sufficient fluency in written English, French, Spanish or Simplified Chinese to be able to complete the patient reported outcome (PRO) questionnaires and use written therapy materials (i.e., study specific patient hand-outs). Eligibility and retention plans are flexible to avoid exclusion of participants based on co-interventions or complications (e.g., pain management strategies, or prophylactic feeding tube use), thereby supporting our planned subgroup analyses for heterogeneous treatment effects (i.e. Aim 3).

#### Inclusion criteria

1. Adults ≥18 years of age diagnosed with head and neck malignancy
2. RT treatment planned for curative intent
3. Planned to receive external beam radiotherapy dose ≥60 Gy to bilateral neck volumes at participating institution; and
4. Sufficient fluency in written English, French, Spanish or Simplified Chinese to be able to complete the study patient reported outcome questionnaires

#### Exclusion criteria

1. Distant metastasis at enrollment;
2. Prior or planned total laryngectomy;
3. Moderate/severe dysphagia at enrollment per baseline videofluoroscopy DIGEST grade ≥ 2 (as graded per central laboratory review),
4. Previously seen by speech language pathologist for swallowing therapy for the current head and neck cancer
5. Diagnosis of second primary non-head and neck cancers in the thorax or the central nervous system at enrollment
6. Head and neck radiotherapy for thyroid or cutaneous/skin primary tumors, regardless of neck fields.

There will be no purposeful exclusion by age within adult age (18 years of age and older), sex, gender, race, or ethnicity. Instead, the composition of age, women, and minorities will reflect the demographic of subjects with HNC who are treated at participating sites. We will adhere to NIH standards for maintaining and presenting data on self-declared race and ethnicity including two ethnic categories (Hispanic/Latino and Non-Hispanic or Latino) and five racial categories (American Indian or Alaska Native, Asian, Black or African American, Native Hawaiian or other Pacific Islander, and White).

At each participating site, coordinators will screen tumour board lists and radiation treatment schedules weekly to identify potentially eligible patients. Site coordinators will then approach the patient at or after their next standard of care clinic appointment (e.g., with radiation oncology, medical oncology, surgery, or speech language pathology) to obtain consent, after a member of the treatment team has first discussed the trial with the patient. All eligible patients who provide informed consent will be consecutively enrolled to capture a representative spectrum of the target population. A screening log will detail clinicopathologic and demographics of all patients screened, approached, enrolled and randomized to examine the representative nature of the trial, and will be transparently reported in accordance with Consolidated Standards of Reporting Trials (CONSORT) standards [30, 31].

### Screening before randomization

After giving written informed consent, participants fulfilling all eligibility criteria will be enrolled in the study. Participants will only be randomly assigned to the study intervention after screening for no/mild baseline dysphagia using videofluoroscopic swallow (VFS) study digital recordings. The screening step requires independent and blinded central laboratory review of the baseline VFS by trained speech language pathologists. Baseline dysphagia is operationally defined as a DIGEST [32] grade of > 2. Screen failure information will be captured in the electronic database to ensure transparent reporting according to the CONSORT guidance. The minimal data set in the screening log includes demography (age, sex, tumor and treatment plan), screen failure details, and eligibility criteria.

### Study withdrawals

Participants are free to withdraw from participation in the study at any time upon request. Furthermore, a site investigator may discontinue or withdraw a participant from the study for the following reasons: if any clinical adverse event (AE), laboratory abnormality, or other medical condition or situation occurs such that continued participation in the study would not be in the best interest of the participant; or, if intent of treatment changes to palliative. The reason for participant discontinuation or withdrawal from the study will be recorded. Subjects who sign the informed consent form and are randomized but do not receive the study intervention may be replaced. Subjects who sign the informed consent form, are randomized and receive the study intervention, but subsequently withdraw or are withdrawn or discontinued from the study, will not be replaced.

### Study interventions

This 3-arm pragmatic trial will compare RE-ACTIVE swallow therapy (Arm 1) to two hierarchical conditions of PRO-ACTIVE swallowing therapy (Arms 2 & 3). All arms will receive an identical, routine baseline swallowing evaluation with the speech language pathologist. Before RT begins, patients will be randomized to 1 of 3 conditions of swallowing therapy as detailed below (pre-RT is operationally defined as before RT begins up to RT treatment day 3, to allow baseline procedures to occur on RT day 1, if necessary, when patients return to the cancer center for care):

#### RE-ACTIVE therapy (arm 1)

Participants will receive no swallowing therapy sessions from the speech language pathologist unless they develop dysphagia. If and when the following signs and symptoms of dysphagia develop, patients will begin RE-ACTIVE therapy as per the same EAT+EXERCISE protocol in Arm 3. The trigger to initiate swallowing intervention was selected based on input from stakeholders including, patients, family members, and a wide array of clinical providers involved in the treatment of HNC patients. Arm 1 participants will “trigger” when any one of the following conditions are met:

1. Participant reports a common dysphagia symptom (i.e., “choking”) on a standardized PRO screen taken weekly during RT, defined as a score ≥ 6 on the MDASI-HN choke item “difficulty choking on foods or liquids” [33].
2. Participant becomes 100% feeding tube dependent as monitored weekly during RT.
3. Participant develops aspiration pneumonia during RT, as per suspicion by any healthcare team member, that then triggers prescription of an antibiotic.

Participants who develop or endorse any one of the above three trigger conditions will be referred to begin RE-ACTIVE swallowing therapy according to the EAT+EXERCISE model within three RT days of their trigger.

#### PRO-ACTIVE therapy (arms 2 & 3)

All participants randomized to PRO-ACTIVE arms will receive in person swallowing therapy sessions with a speech language pathologist once before RT begins then every 2 weeks during RT (weeks 2, 4, 6) and coached to continue daily independent therapy in between live sessions. The two PRO-ACTIVE arms will adhere to the same frequency of live sessions but differ in the intensity of the exercise. Specifically,

**PRO-ACTIVE “EAT” (Arm 2)** will aim to keep the patient eating challenging foods safely and efficiently through the duration of RT (typically 6 to 7 weeks). Arm 2 represents a low intensity PRO-ACTIVE therapy, not requiring any additional exercise or therapy outside of mealtime.

In contrast, **PRO-ACTIVE “EAT + EXERCISE” (Arm 3)** will include the same therapy as Arm 2 (EAT) *and in addition,* the patient will be trained in swallowing exercises to be executed independently between meals (EXERCISE). Arm 3 represents a high intensity PRO-ACTIVE therapy asking the patient to perform a standardized set of throat exercises outside of mealtime.

All three swallowing therapy interventions require no training or expertise beyond that of a typical provider, namely a speech language pathologist, in a usual care setting. Each arm represents a reproducible version of therapy options that are currently provided in usual care settings in this population [19, 22]. In keeping with pragmatic trial standards [34], co-interventions are not prohibited (e.g., pain management, other non-swallowing behavioral therapies, other swallowing therapies post RT, or other systemic cancer treatment including chemotherapy and surgeries), but will be tracked in the trial database. Likewise, patients with feeding tube in situ at baseline will not be excluded, but will be tracked in the trial database. For the purpose of reproducibility and understanding natural adherence, patients will be asked to log adherence to their swallowing therapy interventions with a simple paper “diary” log.

### Outcome measures

This is a pragmatic RCT. Therefore, effectiveness will be assessed through a series of questionnaires and clinical exams to study swallowing status and swallowing-related QOL. A complete list of study activities is summarized in Table 1. The follow-up schedule is carefully planned to align with the schedule of cancer surveillance visits that patients attend in usual practice setting. We will evaluate patients to 12 months to examine long-term effectiveness. Capture of outcome intervals have been set with wide, but discrete windows to enhance flexibility. In all arms, off schedule visits are allowable should problems arise, as per usual care. Clinical testing specified in the protocol represents what is standard at institutions that have adopted the PRO-ACTIVE models as routine care.

**Table 1:** PRO-ACTIVE Schedule of Activities

	Pre-RT		During RT						Post-RT	
	Baseline		Week 1 ±3 tx days	Week 2 ±3 tx days	Week 3 ±3 tx days	Week 4 ±3 tx days	Week 5 ±3 tx days	Week 6 ±3 tx days	3 Months ±2 weeks	12 Months ±4 weeks
**Time**	**1**	**2** (randomization to 3 tx days after RT Start)	**3**	**4**	**5**	**6**	**7**	**8**	**9**	**10**
**Assessments (by chart abstraction and at clinic visits) and Protocol Procedures**										
Informed Consent	X									
Eligibility verification	X									
VFS^a^	X								X	X
Trismus	X								X	X
Randomization^b^		X								
Demographics	X									
Medical History^c^	X							X	X	X
BMI	X			X		X		X	X	X
PSS-HN^d^	X			X		X		X	X	X
RT plan archive^e^								X^i^		
AESI				X		X		X	X	X
Pain Medications	X							X	X	X
Trigger Review**			(X)	(X)	(X)	(X)	(X)	(X)		
**Swallowing Therapy**										
Swallowing Therapy Sessions^f^		X		X		X		X		
**Patient Reported Outcomes (does not require clinic visits)**										
Trigger PRO***			(X)	(X)	(X)	(X)	(X)	(X)		
MDADI	X							X^i^	X	X
Tube PRO	X			XX		XX		XX	X^j^	X^k^
MDASI-HN	X			X		X		X	X	X
Exercise Diary			X*	X*	X*	X*	X*	X*	X	X
MOD^g^	X								X	X
Self-Report Pneumonia^h^	X								X	X
CBI-I	X								X	X
EQ-5D-5L	X								X	X

(X) Only for untriggered RE-ACTIVE arm participants

XX Only for Triggered RE-ACTIVE Arm 1, PRO-ACTIVE EAT and PRO-ACTIVE EAT + EXERCISE Arm 3 participants

X* Only for Triggered RE-ACTIVE Arm 1 and PRO-ACTIVE EAT + EXERCISE Arm 3 participants

^a^ X-Ray involved. Requires central lab review

^b^ randomization completed within 72 h of eligibility verification by completion of central VFS review

^c^ Per chart abstraction by coordinator; if missing in chart, by coordinator contact

^d^ Validated clinic interview along with study specific questions administered by SLP/Clinician (or by a research coordinator if no clinic visits or missed in clinic)

^e^ RT plan DICOM archived to central database

^f^ Conducted by Speech Language Pathologist and only for participants assigned to PRO-ACTIVE arms. Participants in RE-ACTIVE arms who are triggered will begin swallowing therapy according to the EAT-EXERCISE model in arm 3 (PRO-ACTIVE EAT + EXERCISE) within 3 treatment days

^g^ to be completed at select sites only

^h^ administered as part of MOD

^i^ completed at the end of RT

^j^ to be completed every 2 weeks for first 3 months after RT within ±3 days

^k^ to be completed monthly within ±7 days

**Coordinator review of trigger status of RE-ACTIVE participants

***Participant review of PRO dependent trigger items (i.e. tube feed and MDASI #16 choke item)

#### Primary outcome

The primary endpoint of duration of feeding tube dependence was selected because of its high relevance and clear importance to both patients and providers [35, 36]. Maintaining the ability to swallow foods and liquids is the primary functional priority ranked by HNC patients both before and after their treatment [35, 37]. Because feeding tube utilization requires greater resource burden, this endpoint is also relevant to administrators and payers who make decisions about whether to support a healthcare service by way of reimbursement, staffing, or endorsement as best practice. Feeding tube dependence is also pragmatic as a primary endpoint because it is universally measured in usual care, that is, providers ask about and document feeding tube status at routine clinical visits. Therefore, our primary endpoint of feeding tube use can be ascertained by medical chart, in clinic, or by our study specific electronic PRO (ePRO) remotely. A participant will be considered lost to follow-up if feeding tube use cannot be ascertained by any means at 12 months post RT.

#### Secondary outcomes

Secondary outcome measures represent both clinician-graded and patient reported outcome (PROs) measures. These measures were selected based on feasibility [38] at all sites, psychometric validation, familiarity to the investigative team, and/or wide used in routine settings of care, thereby maintaining pragmatic standards. The secondary outcomes are the following:

1. The MD Anderson Dysphagia Inventory (MDADI)

MDADI is a widely used PRO instrument to evaluate swallowing-related QOL in HNC patients [39]. The 20-item questionnaire quantifies an individual’s global, physical, emotional, and functional perceptions of swallowing ability. MDADI was internally developed and validated at MD Anderson Cancer Center and has been integrated in the MDACC dysphagia clinic for more than 10 years. Questionnaire development included engagement of providers and patients with HNC who experienced dysphagia iteratively throughout conception and refinement of the final scale. In a validation sample of 100 patients with HNC, concurrent validity was moderate by comparison with the Performance Status Scale-Head and Neck (Spearman correlation, 0.47-0.61). Correlation with the subscales of SF-36 demonstrated convergent and divergent validity of the MDADI. Test-retest reliability (physical, 0.86; emotional, 0.88; functional, 0.88) and internal consistency reliability (overall Cronbach’s alpha, 0.96) were sound [39]. Subsequent validation studies further demonstrated suitable psychometric properties in patients with different ethnic-cultural backgrounds [40–42]. Perceived dysphagia as quantified by the MDADI is time-dependent, radiotherapy dose-dependent and varies by extent of surgery and tumor site in HNC samples [35, 43]. The investigators have previously shown a 10-point between-group difference to discriminate a clinically meaningful difference in swallowing outcome [44].

2) The MD Anderson Symptom Inventory for Head and Neck Cancer (MDASI-HN)

MDASI-HN is a multi-symptom PRO instrument designed to measure severity or burden of cancer-related symptoms and their interference with or effect on patients’ daily functioning [33, 45]. Symptom burden refers to the severity of physical or mental experience by a patient with a specific symptom. Symptom measures are more specific and direct than QOL measures and thus are more sensitive to treatment-related changes. The 28-item MDASI-HN multi-symptom inventory measures include 13 core items (“systemic symptoms”: pain, fatigue, sleep, etc.), 9 head and neck specific items (“local symptoms”: dry mouth, mucus, shortness of breath, taste, etc.), and 6 interference items (activity, work, relations, etc.). The core MDASI items were validated for use in cancer patient populations regardless of the specific diagnosis or type of therapy [45] and thus can be used to compare symptom burden between different types of cancer. The 9 head and neck specific items were validated internally at MDACC with regard to construct and concurrent validity in a cohort of 205 HNC patients [33]. Internal consistency reliability is high in the core MDASI score, the 9 head and neck-specific items, and the 6 interference items (Cronbach’s α: 0.72–0.92). MDASI-HN can be completed in less than 7 min. MDASI-HN offers quantification of overall symptom burden, but also 2 dysphagia-specific items (“difficulty swallowing”, “choking”) and will be used to understand and adjust for symptoms such as mouth sores, pain, or dry mouth, that make eating challenging during RT. The investigators have experience in using MDASI-HN in weekly radiation clinics for symptom monitoring with a resultant publication characterizing the evolution and cumulative increase in symptoms weekly during HNC RT [46]. Symptom burden can be considered both a secondary outcome, but also a potential confounder (i.e., competing toxicity) that may influence adherence to swallowing therapy.

3) Videofluoroscopic Swallow (VFS) Studies

The VFS is a dynamic, radiographic imaging study, in which the patient is administered radiopaque labeled bolus volumes in a series of standardized liquid and solid food consistencies to examine oropharyngeal swallow physiology, aspiration, and/or pharyngeal residue. VFS is considered a gold standard measure of dysphagia in clinical practice and is routinely conducted by speech language pathologists in medical settings*.* Digital videos from VFS will be scored centrally by a trained speech language pathologist blinded to clinical history using the following measures: DIGEST [32], pharyngeal constriction ratio (PCR) [47], penetration-aspiration scale (PAS) [48] and maximum pharyngoesophageal segment opening (PESmax) [49]. DIGEST, the primary measure to determine study eligibility, was developed and validated by the study team [32] as a CTCAE compatible VFS-derived severity grade of pharyngeal dysphagia. DIGEST is reliable (intra- and inter-rater weighted *k* 0.82–0.84 and 0.67–0.81, respectively) and discriminates pharyngeal pathophysiology (MBSImP™©:*r* = 0.77, *p* < 0.001), perceived dysphagia (MDADI: *r* = − 0.41, p < 0.001), and diet (PSS-HN: *r* = − 0.49, p < 0.001) in HNC survivors [32]. DIGEST is sensitive to change in swallowing function over time [50]. PCR, the primary endpoint of VFS for secondary analysis (**Aim** 2), is a continuous measure of pharyngeal swallowing strength. PCR is a pixel based measure computing area of the pharynx at maximum constriction at peak swallow over the resting area of the pharynx from the mid-sagittal radiograph. PCR provides a reliable radiographic surrogate measure of pharyngeal strength (measured against gold standard pharyngeal manometry) [47, 51, 52] and discriminates aspiration status in dysphagia populations [52]. PAS is an 8-point ordinal classification of swallow airway safety capturing bolus penetration and aspiration, where a score of 1 indicates no airway entry, 2–5 indicates penetration, and 6–8 indicates aspiration. PAS ratings are highly reliable (ICC for inter- and intra-rater reliability: 0.96 and 0.95–0.97, respectively) and predictive of aspiration pneumonia after chemoradiation (*p* < 0.001, AUC = 0.72) [1]. PESmax provides a reliable radiographic surrogate marker of stricture at the level of the cricopharyngeal muscle, which if present will critically disrupt transport of the bolus into the esophagus [53].

4) Performance Status Scale Head and Neck (PSS-HN)

The PSS-HN [54] is a 3-item validated semi-structured interview tool capturing normalcy of diet, public eating, and, understandability of speech. Each item is scored from 0 to 100, with the higher score indicating better performance. The PSS-HN correlated well with the FACT-HN, especially for normalcy of diet and eating in public (r = 0.66, *p* < 0.0001; r = 0.42, p < 0.0001, respectively) and is a reliable tool, sensitive to differences in functioning when applied to HNC patients [55]. The PRO-ACTIVE trial adaptation of PSS-HN includes additional questions regarding tracheotomy status and feeding tube utilization.

5) Medical Outcome of Dysphagia (MOD)

The Medical Outcomes of Dysphagia (MOD) is a newly developed 3-part inventory that targets patient reported symptoms related to health consequences secondary to dysphagia, namely: malnutrition (MOD-Nutrition), aspiration pneumonia (MOD-Pulmonary) and mood changes such as depression and anxiety (MOD-Psychology) [56]. The MOD-Nutrition sub-scale consists of 13 items, of which all but one have either a 3, 4 or 5-point ordinal response; the remaining item quantifies food intake. The MOD-Pulmonary sub-scale consists of 11 items, of which all have a 5-point ordinal response. Likewise, the MOD-Psychology sub-scale consists of 45 items, of which all have a 5-point ordinal response. The study assessing reliability and divergent validity of the items in the three MOD subscales has recently been completed, and included over 350 patients, among whom 77% had a diagnosis of dysphagia secondary to head and neck cancer. Each of the sub-scales has excellent inter-rater reliability with the following total score ICC (95% confidence interval): MOD-Nutrition, ICC = 0.84 (0.73–0.91); MOD-Pulmonary, ICC = 0.89 (0.81–0.94); and, MOD-Psychology ICC = 0.90 (0.83–0.94). The items on the MOD were derived from an extensive review of the literature combined with collective opinion of clinicians, patients and caregivers [56].

6) Cancer Behavior Inventory – Version 3 (CBI-V3)

The Cancer Behavior Inventory was developed as a comprehensive measure of self-efficacy strategies for coping with cancer, drawing from self-regulation and self-efficacy theories [57–59]. The most recent version of 27 items has undergone extensive revision to ease administration and strengthen psychometric properties. The measure is designed at a grade 6 reading level and has 7 subscales or factors including Maintaining Activity and Independence, Seeking and Understanding Medical Information, Emotion Regulation, Coping with Treatment Side Effects, Accepting Cancer/Maintaining a Positive Attitude, Seeking Social Support, and Using Spiritual Coping. Internal consistency is reported as Cronbach’s alpha of .946, and test-retest reliability as *r* = .890 (4 months) with mixed groups of cancer patients [60]. Validity coefficients indicate strong psychometric properties with higher scores on the CBI associated with better adjustment to cancer, higher quality of life, and lower levels of emotional distress. Each item presents an activity a cancer patient might do during and after cancer treatment and is scored on a Likert type scale ranging from 1 (*‘not at all confident’*) to 7 (‘*totally confident’*). Scores are derived for each of the subscales as well as an overall total score.

7) EQ-5D-5L

EQ-5D-5L is a modified version of the widely used EuroQOL 5 Dimension [61]. The brief 6-item EQ-5D-5L questionnaire is used to describe and value health. The EQ-5D-5L descriptive system measures five levels of severity in each of the existing five dimensions (mobility, self-care, usual activities, pain/discomfort, and anxiety/depression). The questionnaire also contains a Visual Analog Scale, by which respondents can report their perceived health status with a grade ranging from 0 (the worst possible health status) to 100 (the best possible health status).

8) Medical History

Intake details, radiation therapy details, systemic therapy/chemotherapy details, tumour status, surgery history, tracheotomy, feeding tube insertion, removal, and use dates, feeding tube dependence, concomitant swallowing therapies, vital status, BMI, and complication details will be collected by review of patient medical record at specified intervals.

9) Pain Medications

All pain medications, including over-the-counter medications, reportedly taken in the past 48 h are noted on the pain medication form in the case record form (CRF) at pre-specified intervals (e.g. baseline, week 6 of RT, 3 months and 12 months post RT).

10) Mouth Opening/Trismus

Interincisal opening will be measured at baseline, and 3 months and 12 months post RT using a standardized TheraBite® Range of Motion Scale (TRMS) [62]. Reference points will be noted (gingiva and/or teeth) on the study case report form.

11) Hospitalization/ Emergency Department Presentation

Hospitalization and/or emergency department presentation will be abstracted from the medical record and by asking participants to identify such events to any external institutions anytime during their participation in the trial.

### Safety and adverse events

The therapy provider and/or study team will review adverse events of special interest (AESI) at each study visit. Two adverse events of special interests (AESIs) in Common Terminology Criteria for Adverse Events, CTCAE version 5 [63] monitored by the DSMB (Data Safety Monitoring Board) for this trial safety include: weight loss of grade > 2, or aspiration of grade > 2. In addition, at week 6 of RT, the study team will record the onset and peak severity of selected adverse events during RT, also graded using CTCAE version 5, for the following categories: dysphagia, oral and pharyngeal mucositis, oropharyngeal pain, nausea, vomiting and dermatitis. Attribution of each AESI to study intervention will be assessed by the clinician and/or study team according to best clinical judgment. It is expected that many of the AESIs will be related to the RT (cancer treatment). The summaries of AESIs will be reviewed regularly by the trial DSMB.

### Data management plan

The data management plan was developed in accordance with PCORI Methodology Standards for Data Integrity and Rigorous Analysis*.* Data sources include the electronic health record, PRO questionnaires, imaging data (videofluoroscopy and DICOM radiation dose-grids), and study specific questionnaires. A robust centralized web-based database was designed and maintained by the Techna Institute: Health Informatics Core, an independent research institute within UHN. Manuals of Procedures (MOP) will ensure harmonized entry of individual data items from each source and site under a common data model structure. Authentication, anonymization, and confidentiality plans are detailed in the contractual arrangement with the Techna Institute.

### Statistical analysis

#### Primary endpoint

For the primary endpoint, an intention-to-treat (ITT) approach will be applied incorporating a gate-keeper method to control risk of Type 1 error in the setting of multiple comparisons [64, 65]. The primary hypothesis (Hyp 1.1) is that the combined PRO-ACTIVE interventions (PRO-ACTIVE EAT and PRO-ACTIVE EAT+EXERCISE) will result in a better swallow outcome compared to RE-ACTIVE as measured by duration of feeding tube use in the first year after the completion of RT. If PRO-ACTIVE is superior in Hyp 1.1, we will assess the dose response of the PRO-ACTIVE arms. The secondary hypothesis (Hyp 1.2) is that the most intensive intervention (PRO-ACTIVE EAT+EXERCISE) will be more effective than the least intensive (PRO-ACTIVE EAT).

The initial analysis will compare the RE-ACTIVE group to the combined PRO-ACTIVE groups in terms of the duration of feeding tube use operationalized as count of days of tube use from end of RT to last tube use within the 12-month study period by means of a multiple linear regression model with covariate adjustment. The presence of a feeding tube which is not being used for feeds is disregarded. The treatment effect will be expressed as absolute mean difference along with 95% confidence intervals. If this test yields *p* < 0.05, the two PRO-ACTIVE groups (EAT versus EAT+EXERCISE) will be compared using the same linear analysis method. The meaningful variables we will include in the regression model are: age (as a continuous variable); HPV status (oropharyngeal site vs all others); surgery (yes/no); chemotherapy (yes/no); radiation dose (as a continuous variable); and, facility (as a categorical variable to account for practice variation across the eleven sites).

The largest threat of missing data is non-random loss of outcomes data for the primary endpoint. Missing data for the primary endpoint is extremely unlikely because it is a simple variable that can be ascertained in clinic or remotely via telephone/web interview of the patient. Non-random missing data is more likely for outcomes requiring clinic examination (i.e., videofluoroscopy) for which we have estimated attrition at 26% based on prior longitudinal work from the investigators [38]. For outcomes missing in > 10% of patients, we will consider multiple approaches. One method we may employ is inverse probability weighting approach to account for statistical uncertainty due to missingness. Another option we will explore is multivariate imputation using chained equations [66]. When similar inferences are derived from complete case analysis and imputation, we will report complete case analysis results for simplicity and transparency.

#### Secondary endpoints

The analysis of the secondary endpoints is not dependent on the findings of the primary endpoint. The hypothesis (Hyp 2.1) is that the combined PRO-ACTIVE interventions will result in a better secondary outcomes including videofluoroscopic swallowing evaluations, functional status measures, health status measures, and patient reported outcomes 3 months following the completion of RT. For the secondary outcomes, ITT analysis will again use a gate-keeper approach to assess differences between arms. Continuous outcomes will be evaluated with ANCOVA approach (multiple linear models regressing outcome on randomization arm plus the baseline measure for adjustment) at the 3-month post-treatment interval when a baseline measurement is present and a t-test (or suitable replacement) otherwise. The treatment effect will be expressed as the mean adjusted difference, or equivalently, mean adjusted change along with 95% confidence intervals. Binary outcomes will be compared by means of a chi-square test or Fisher’s exact test if expected counts are less than 5 and treatment effects will be expressed as risk differences with 95% confidence intervals. Sensitivity will be assessed in terms of consistency of inference as well as magnitudes of effect size using distributional measures of Minimal Clinically Important Difference (MCID) [67] or anchor-based MCID [44] when available to interpret whether observed differences are meaningful. Exploratory analyses will be conducted on all outcomes with repeated measurements using mixed-effect models to examine time by treatment interactions. Exploratory subgroup analyses will examine the relative benefit or harm of the PRO-ACTIVE and RE-ACTIVE swallowing interventions in relevant subgroups according to the following: patient (age, tumor site, prior surgery); and provider (radiation dose, chemotherapy use, prophylactic feeding tube use).

#### Sub-group analyses (heterogeneous treatment effects, HTEs)

Acknowledging that certain groups may benefit substantially while others may benefit far less, exploratory heterogeneous treatment effect (HTE) analyses are planned (Aim 3). The goal of the HTE analyses is to explore if there are subgroups for whom swallowing interventions have varied impact. For hypothesis-generating analyses, HTEs will be assessed by testing the treatment arm by subgroup interaction. Analyses are planned according to two potential sources of HTE [34]: the patient and provider**.** For parsimony, a likelihood ratio test will examine proposed HTEs simultaneously. Irrespective of their significance, the subgroup effects will be reported (with 95% confidence intervals) to be assessed in terms of clinical significance.

We have identified several participant subgroups for whom differences in treatment response or swallowing outcomes may exist. Preliminary data suggest superior swallowing outcomes in younger patients [10], those with HPV-associated cancers [68–70] those with no prior surgery [68], those who receive lower doses of RT [10], those who avoid chemotherapy [68, 71] and who avoid prophylactic feeding tube placement [9]. Each of these classifications will be examined as relevant subgroups in the exploratory HTE analysis, as justified below:

### Sample size calculation

Sample size calculations, based on a 2-sample t-test, assume 952 patients enrolled with 1:4 allocation to RE-ACTIVE (Arm 1, *n* = 190) and PRO-ACTIVE arms (Arms 2 and 3, *n* = 381 per group). The attrition factor is set at 5% because the primary endpoint can be ascertained without clinical encounter. Thus, the resultant sample size estimated is 180 in Arm 1, 361 in Arm 2, and 361 in Arm 3. For the primary comparison (Hyp 1.1) of Arm 1 (*n* = 180) versus the combined Arms 2 and 3 (*n* = 722), assuming ITT analysis with type 1 error probability of 0.05 (two-sided) yields 80% power to detect a small effect size of .23 SD for duration of feeding tube use. For the analysis of Hyp 1.2 in the gate-keeper approach, we will compare intensity of PRO-ACTIVE therapies (Arm 2 versus Arm 3, *n* = 361 per group), assuming type 1 error probability of 0.05 (two-sided), we have 80% power to detect again a small effect size of 0.21 SD for the primary endpoint of feeding tube duration. We expect the t-test will not be sufficient as the primary analysis given it does not allow for covariate adjustment. Therefore, we will apply the multiple linear regression model as the primary analysis for Aim 1. The power calculation conducted assuming t-tests for analysis of hypotheses 1.1 and 1.2 and deriving a sample of 952 patients remain sufficient for the linear model because by adjusting for variables associated with the mean response, the distribution of the residuals is expected to improve. Therefore, the power for the same hypothesized difference in the primary outcome attributed to treatment in the regression framework will be at least as much as for that for the t-test.

For the secondary analysis (Aim 2) in the gate-keeper approach, we will compare intensity of PRO-ACTIVE therapies (Arm 2 versus Arm 3, *n* = 361 per group), assuming type 1 error probability of 0.05 (two-sided), we have 80% power to detect again a small effect size of 0.24 SD for PROs and VFS measures. The attrition factor for the secondary analysis is set at 26% because they are more prone to attrition due to required clinical visits and survey responses required from participants. As an exception, feeding tube dependence at 3-months uses an attrition factor of 5%, for the same rationale as the primary endpoint.

## Discussion

Despite continued improvements in RT techniques to optimize functional outcomes by minimizing collateral normal tissue damage [10], almost all patients experience some degree of early dysphagia. Dysphagia is not only an acute toxicity but a progressive and chronic issue particularly relevant to the rapidly growing pool of younger HNC survivors who achieve long-term cure [24].

Several single institution observational and controlled efficacy trials as well as two recent systematic reviews have shown benefits of swallowing therapy, regardless of its timing and intensity, to various outcomes, including feeding tube dependence, QOL, swallow efficiency, function, and swallow physiology [21, 23]. While offering a strong evidence base for efficacy of swallowing interventions in this population, these small efficacy trials and meta-analyses were unable to identify the timing (PRO-ACTIVE versus RE-ACTIVE) or intensity (less versus more) that is most effective in treating dysphagia. As such, the PRO-ACTIVE trial was designed as a pragmatic, real-world examination to compare effectiveness of 3 models of swallowing therapy during the radiotherapy interval.

### Strengths and limitations

Stakeholder engagement in the development and implementation of the PRO-ACTIVE trial is a major strength. In keeping with the PCORI standards, stakeholder consultation and collaboration was built into all study phases, from conception through dissemination. As an initial step in engagement, the trial concept was presented to the National Foundation on Swallowing Disorders (NFOSD) who endorsed the relevance of the *decisional dilemma.* Provider stakeholders in the investigators’ Radiation-Associated Dysphagia Investigative Working Group also met over 30 h to refine proposed Arms, pragmatically align methodologic decisions, and to enhance feasibility of study procedures across different geographic and sociodemographic settings. Stakeholder panels in both Canada and the USA share governance and consultation over the life of the study. Diverse stakeholders representing the perspectives of patients, caregivers, physicians, allied health care providers, policy makers, and payors convene in these panels at regular intervals to advise the investigators in key trial issues. Stakeholder engagement has informed operationalization of the ‘trigger’ criteria for the RE-ACTIVE therapy arm, secondary outcome measurement, recruitment/retention strategies, and the content of study-specific patient educational materials.

PRO-ACTIVE was designed in accordance with pragmatic trial standards. The investigators used the PRECIS-2 tool [27, 28] to guide development of the trial protocol to ensure that findings are derived from a study design that is reflective of, and thus generalizable to, the real-world setting. For this reason, outcome measures represent those most practically implemented in the clinical setting. A limitation of the trial protocol includes the omission of detailed RT dose data, specifically including the dose distributions to normal tissues (commonly referred to as organs-at-risk, or OARs). While it is outside the scope of the PRO-ACTIVE trial protocol to analyze RT plans to extract OAR dose, the trial protocol includes the upload and archiving of the full RT DICOM data set including the anatomic imaging and dose files. This RT plan archive will allow the investigators the opportunity for future post-hoc correlative analysis of RT plans with other trial data.

Other key omissions from the trial protocol include details of financial burden or cost incurred by patients and their caregivers. The investigators recognize the critical importance of this issue in modern healthcare. This domain was outside the scope of allowable funding for the PRO-ACTIVE trial. The investigators intend to consider alternate funding sources to examine cost in a subset of PRO-ACTIVE trial participants for exploratory or hypothesis-generating purposes. Another avenue for future or related work is qualitatively exploring the perspective of patients, caregivers, and providers in the lived experience of swallowing therapy during the PRO-ACTIVE study.

The data obtained from PRO-ACTIVE is intended to help address the decisional dilemma about which type and timing of swallowing therapy provides the best outcomes in the real world and in distinct subgroups of HNC survivors. Given the minimal risk associated with participation, there is great potential benefit from this study to improve quality of care and QOL in HNC survivorship. Also, since all participants enrolled in PRO-ACTIVE will receive a form of swallowing therapy, there is potential for individual benefit in all arms. There is currently insufficient evidence to guide decisions of best swallowing therapy options in real world settings. Once clinically manifested, radiation-associated dysphagia is difficult to reverse. Real world comparisons of common therapies represent a critical step forward to improve quality of care in the health care system and the QOL of individual patients. These data might also translate to better health by way of improved nutritional status and diminished risk of death from aspiration pneumonia. The potential yield is far greater than the minimal risk imposed upon patients enrolled to the trial.

## Data Availability

not applicable.

## Abbreviations

AE: Adverse Event
AESI: Adverse Events of Special Interest
ANCOVA: Analysis of Covariance
CONSORT: Consolidated Standards of Reporting Trials
CRF: Case Report Form
DCC: Data Coordinating Center
DSMB: Data Safety Monitoring Board
ED: Emergency Department
eCRF: Electronic Case Report Forms
HNC: Head and Neck Cancer
IRB: Institutional Review Board
ITT: Intention-To-Treat
MCID: Minimal Clinically Important Difference
MDACC: MD Anderson Cancer Center
MOP: Manual of Procedures
NCT: National Clinical Trial
NIH: National Institutes of Health
PI: Principal Investigator
PM: Princess Margaret Cancer Centre
PRO: Patient Reported Outcomes
QOL: Quality of Life
REB: Research Ethics Board
RT: Radiotherapy
SOA: Schedule of Activities
UHN: University Health Network
US: United States
VFS: Videofluoroscopic assessment of swallow
